# Practical Dietetics, with Reference to Diet in Disease

**Published:** 1907-04

**Authors:** 


**Practical Dietetics, with reference to Diet in Disease.** By **Alida Frances Pattee,** Late Instructor in Dietetics, Bellevue Training School for Nurses, New York. Fourth edition, 12mo, 300 pp. New York: A. F. Pattee, Publisher, 52 West 39th Street. (Price, $1.00 net).

Here is a little book with an enormous amount of solid, practical, everyday information within its covers; a book which is well-nigh invaluable to the nurse and one which will be of really inestimable benefit to the physician. In a word it is a most excellent guide for proper diet in sickness, covering as it does the correct preparation of food for the invalid and the convalescent. In detail it gives the methods which experience has shown to be the best for preparing and administering foods in liquid, semisolid and solid states. There are also diet lists with especial references to the things to be avoided in given cases, with instructions for the intelligent dieting of infants and children. A splendid feature is the diet list used by eminent medical men and some of the more prominent hospitals in this country. Typical of the character of the book is its binding, the blue and white of the nurse's uniform.

